# Quantifying the Effect of Financial Burden on Health-Related Quality of Life among Patients with Non-Hodgkin’s Lymphomas

**DOI:** 10.3390/cancers12113325

**Published:** 2020-11-11

**Authors:** Richard Huan Xu, Eliza Lai-yi Wong, Yi Su, Hongyu Zhang, Wei Zhang, Dong Dong

**Affiliations:** 1 Jockey Club School of Public Health and Primary Care, Faculty of Medicine, The Chinese University of Hong Kong, Hong Kong, China; richardhxu@cuhk.edu.hk (R.H.X.); lywong@cuhk.edu.hk (E.L.-y.W.); 2 Centre for Health Systems and Policy Research, The Chinese University of Hong Kong, Hong Kong, China; 3 Department of Health Affairs, Jinling Hospital, School of Medicine, Nanjing University, Nanjing 210000, China; sy_njzy@126.com; 4 Department of Hematology, Peking University Shenzhen Hospital, Shenzhen 518000, China; hongyuzhang@pkusz.com; 5 Department of Hematology, Peking Union Medical College Hospital, Beijing 100730, China; vv1223@vip.sina.com; 6 Shenzhen Research Institute, The Chinese University of Hong Kong, Shenzhen 518000, China

**Keywords:** non-Hodgkin’s lymphomas, health-related quality of life, financial burden, cancer

## Abstract

**Simple Summary:**

Financial burdens result from the growing out-of-pocket costs associated with cancer care to help patients regain physical and psychological health-related quality of life (HRQoL) are dramatically intensified. The aim of our study was to assess the association of HRQoL with financial burden using both subjective and objective methods among patients with non-Hodgkin’s lymphoma (NHL) in China. A majority of the patients reported suffering moderate to high financial burdens. A significant relationship between increased financial burden and reduced HRQoL was identified. Patients tended to report a poorer HRQoL when using objective method than using subjective method to estimate financial burden. Medical professionals should involve patients and their families into the clinical decision making and provide them cost-effective plans.

**Abstract:**

Objective: This study aimed to assess the association of health-related quality of life (HRQoL) with financial burden among patients with non-Hodgkin’s lymphoma (NHL) in China. Methods: The data used for the analyses came from a nationwide survey to investigate the health status of patients with lymphomas in China. The EQ-5D and EORTC QLQ-C30 were used to assess the patients’ HRQoL. The financial burden was calculated using both subjective and objective methods. The chi-squared test, Kruskal–Wallis one-way analysis of variance, ordinal least squared model, and Tobit regression model were used to estimate the relationship between financial burden and HRQoL. Results: Data from 1549 patients who reported living with 11 subtypes of NHL were elicited for our analysis. Approximately 60% of respondents reported suffering moderate to high financial burdens. A significant relationship between increased financial burden and reduced HRQoL scores, including the EQ-Index, physical, emotional, and social functioning, was identified. Compared with using an objective method to measure financial burden, patients with NHL indicated a poorer HRQoL when using a subjective method to measure financial burden. Conclusion: Medical professionals should select highly cost-effective treatments and ensure that patients understand the potential financial consequences of those treatments.

## 1. Introduction

Non-Hodgkin’s lymphoma (NHL), which includes more than 60 subtypes, is a cancer that originates in the lymphatic system and spreads through the system in a non-orderly manner [1]. NHL accounts for nearly 90% of lymphomas and is listed as the ninth and tenth most common types of cancer for males and females worldwide [2]. In Asian countries, the incidence rate of NHL was listed as eighth, ninth and thirteen most common types of cancer in Singapore, South Korea and Japan, respectively [3]. In China, NHL is the tenth most common cause of cancer-related deaths and has an incident rate of 6.43/100,000 [4]. NHL leads to a great number of negative impacts on patients’ health-related quality of life (HRQoL) because of its chronicity, symptomology, and complications.

HRQoL is an important outcome of cancer care, which can reflect an individual’s perceived physical and mental health over time. HRQoL synthesizes the patients’ preferences and needs with the medical professionals expertise, which are necessary parts of evidence-based decision making in both clinical medicine and public health [5]. In recent years, the increased incidence of cancer, combined with the overall decline in its mortality rate has resulted in a growing number of cancer survivors. The extended life spans mean that cancer patients have the opportunity to focus on HRQoL beyond pure survival. However, because most survivors are likely to live with chronic HRQoL impairments, such as functional impairment, depression and isolation [6,7], the corresponding costs increase as new, advanced, and expensive treatments and medicines are adopted as standards of cancer care to maintain and improve their HRQoL [8]. As cancer patients live longer, the greater expenses for longer-term care is incurred. Financial burdens result from the growing out-of-pocket (OOP) costs associated with cancer care to help patients regain physical and psychological HRQoL are dramatically intensified.

Studies specifically examining the relationship between financial strain and HRQOL during and after cancer care have gained increased interest and attention [9,10,11]. In a recent systematic review, almost half of the patients in the United States (US) reported a poor physical or psychologic HRQoL related to the cost of cancer treatment [12]. Another study indicated that, in the US, people diagnosed with cancer show 2.5-times greater likelihood of declaring bankruptcy, and report greater mortality risk than those who did not [13]. Other studies demonstrated that cancer patients with a high financial burden tend to report poor HRQoL due to more medical adverse events, such as lower adherence to treatment, poorer prognosis, shorter survival time, and greater risk of recurrence [14,15,16]. In light of HRQoL providing a rather comprehensive assessment of the effectiveness of cancer prevention, treatment and prognosis, examining its associations with patients’ financial hardship is valuable and informative in terms of resource allocation decision making.

In China, although most citizens are protected by three basic medical insurance plans provided by the state (the Urban Employee Basic Medical Insurance for employed workers in urban areas, the Urban Resident Basic Medical Insurance for unemployed urban residents, and the New Rural Cooperative Medical Scheme for rural residents) [17], cancer patients, including patients with NHL, are rarely fully protected by these resources [18]. Most NHL treatments are not covered by basic medical insurance plan, and therefore, patients have to suffer extremely high OOP payments. Meanwhile, even though part of NHL-related costs can be reimbursed by some other commercial insurance plans, lost ability to work due to the disease, transportation costs to get treatment, and impact on spouse’s income can aggravate the patient’s vulnerability to poverty and lead to a worsened HRQoL [19]. Nevertheless, to date no systematic assessment of the relationship between financial burden and HRQoL among patients with NHL in China.

When estimating the cancer-related financial burden, both subjective and objective methods have been adopted in previous studies [16,20,21,22]. The objective burden directly stems from the OOP expenses of cancer care, e.g., surgeries, drugs and other medical services [23]. It is highly likely to be aggregated with the deterioration of the patients’ health. Subjective burden is the consequence of cost concerns by the individual cancer patient. It could be the result of the accumulation of OOP expenses, the reduction of the household’s wealth, and worry about the effectiveness of treatment available to and used by the patient [24]. Although they can provide valuable insights from different perspectives to facilitate decision-making in affordable drug pricing and insurance model development, both objective and subjective methods have some limitations. For example, the subjective method might be subject to limitations such as the way the question asked and the individual’s health status on the survey day [16]. However, the objective method is highly likely to lead to an over- or underestimation of the cost due to recall bias [25]. Given the rising medication prices, health insurance costs, and other disease management expenses of NHL in recent decades, some hidden costs may not be directly measured by using a single method [26]; it is increasingly encouraged that a composite perspective is to assess financial hardship among patients with NHL. Thus, the aim of this study was to estimate the relationship between HRQoL and financial burden using both subjective and objective methods among patients with NHL in China.

## 2. Results

The respondents’ characteristics are presented in Table 1. A total of 52.2% of respondents were males, and the mean age was 43.3 years. Approximately 90% of respondents completed secondary or above education, half of them reported being fully employed, and approximately 88% patients had been living with NHL for less than six years. Nearly half of the respondents reported suffering from severe objective financial stress after they completed NHL-related treatment. A total of 11 subtypes of NHL were identified; approximately 37.6% reported having diffuse large B-cell lymphoma, whereas 1.7% reported having Burkitt lymphoma. Compared with patients with NHL who reported living with low financial burden, those with high burden were more likely to be less educated, to be uninsured, living in rural areas, and to have low income.

Table 2 presents the proportion of respondents who reported having subjective/objective financial burden, stratified by sex. Approximately 42.4% and 39.4% of respondents reported living with low financial burden using objective and subjective methods, respectively. When respondents reported having moderate financial burdens, the difference (10.8%) between the subjective and objective measures was largest. More female (42.4%) respondents reported having a lower financial burden than males (36.8%) when using the subjective method. However, when using the objective method, the results were reversed: compared with females (41%), more males (43.7%) reported having a low financial burden. The overall agreement rate between objective and subjective methods was 44.8%, where the rate for female respondents (50.3%) was slightly higher than that for male respondents (44.7%).

**Figure 1:**
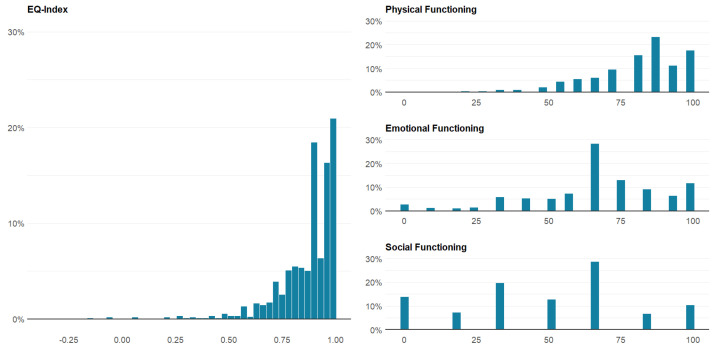
The score distribution of HRQoL measures.

**Figure 2:**
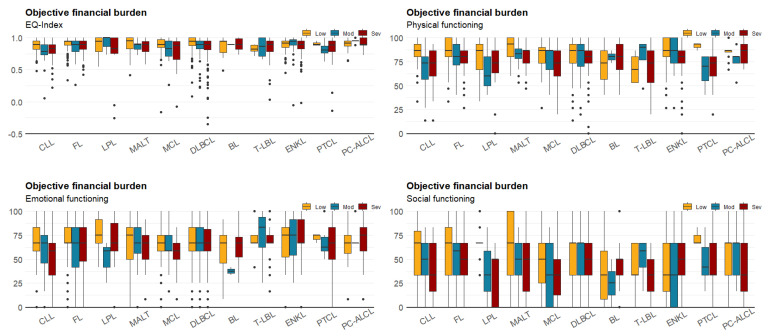
Boxplot for patients’ HRQoL and objective financial burden.

**Figure 3:**
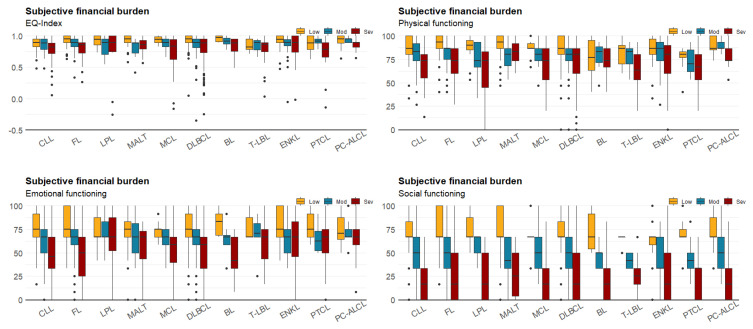
Boxplot for patients’ HRQoL and subjective financial burden.

Table 3 shows the respondents’ HRQoL scores according to the different levels of reported financial burdens. The differences in HRQoL scores between different levels of financial burden were larger when using the subjective method than when using the objective method. For social functioning, the scores dropped by 47.6% from moderate to high financial burden when measured using the subjective method. However, for emotional functioning, the scores increased approximately 0.5% from moderate to high financial burden when measured using the objective method. Mean scores of EQ-Index and other different HRQoL dimensions stratified by treatment status are presented in Table 3. Figure 1 shows the distribution of the EQ-Index and scores of physical, emotional, and social functioning of the QLQ-C30. For the EQ-Index, more than 20% of respondents reported full health (EQ-Index = 1.0), but the values were negatively skewed. For emotional and social functioning, the peak was approximately 65. Boxplot shows the results of HRQoL measures for patients with different subtypes of NHL, and stratified by financial burden (Figure 2 and Figure 3). Overall, patients with severe financial burden showed a poor performance on the HRQoL measures. The trend was more consistent and unambiguous when using the subjective method to estimate the financial burden.

The relationship between HRQoL and financial burden adjusted by patients’ demographics, SES treatment and the duration of NHL is demonstrated in Table 4. Overall, there was an association between decreased HRQoL and increased financial burdens on the basis of using both subjective and objective methods. However, when using the objective method to estimate the financial burden, the reduction in HRQoL scores was larger than when using the subjective method. The decrement of social functioning scores was faster than the decrement of the other HRQoL component scores, regardless of whether they were estimated by subjective (coefficient [β] = −43.6, *p* < 0.001) or objective (β = −8.8, *p* < 0.001) methods (full results are presented in Tables S1–S4).

## 3. Discussions

To date, limited studies have explored the association of financial burden with HRQoL using both subjective and objective methods [27]. To the best of our knowledge, this is the first study to investigate the NHL-related financial burden and its association with HRQoL using a large population-based sample in China. We found that, in this study, although nearly all respondents could receive partial reimbursement from participating in government sponsored health insurance or certain commercial insurance plans, approximately 60% of them reported suffering from moderate to high financial burdens when managing their cancer and cancer-related side effect. A significant association between increased financial burden and decreased HRQoL was identified across domains of individual utility and physical, emotional, and social functioning. Compared with measuring financial burden using the objective method, patients with NHL indicated a poorer HRQoL when using the subjective method to measure their financial hardship.

The link between financial burden for NHL patients and negative mental HRQoL was identified by our study. This is in line with the findings reported by previous studies; for example, Park & Look found that higher levels of financial burden were more strongly associated with lower mental HRQOL than with physical HRQOL [16]. Kale et al. reported that there is a stronger negative association of financial burden with mental health than physical health for cancer survivors [10]. Another US study also demonstrated that the financial burden imposed by illness and treatment affects cancer survivors’ emotional HRQoL [28]. In our study, patients with NHL reported 17% lower emotional HRQoL scores than physical HRQoL scores. It is not surprising that mental problems, such as depressed mood, and worrying about NHL recurrence, often lead to greater worries regarding financial burden, which in turn cause more emotional stress and depression. Schnipper and Varner found that patient’s anxiety about unable to actively control their cancer can peak [29]. However, considering that NHL consists of several subtypes and that healthcare-related spending for each subtype may be diverse, the specific associations should be further clarified.

In this study, we found that, compared with using an objective method to estimate patients’ financial hardship, using a subjective method generated a larger decrement in patients’ mental HRQoL than in their physical HRQoL. This is similar to the findings reported by another study that examined the relationship between HRQoL and financial burden among patients with lung cancer. They found that patients’ mental wellbeing decreased heavily when using the subjective method to calculate the financial burden than when using the objective method [27]. HRQoL is a patient-centered multidimensional concept that usually reflects subjective evaluations of both positive and negative aspects of life over time [30]. Subjective financial burden is a measure that can represent a cognitive average of financial conditions, and it is not unexpected that it is strongly associated with patients’ HRQoL [31]. However, in this study, we used the healthcare cost-to-income ratio to represent the objective financial burden; while it was identified as a simple measure, some bias might be existed. It remains inconclusive whether the subjective financial indicator is a better predictor to reflect the variation of HRQoL than using a more comprehensive ‘objective’ measure. Further studies are needed.

The EQ-5D, as a standard measure for health economic evaluations, is rarely used in studies that focus on investigating the effect of financial burden on patients’ HRQoL. In this study, we reported the association of financial burden with the EQ-Index, a summary score integrating both physical and emotional utility, among patients with NHL. Compared with the Chinese general population (EQ-Index = 0.96) [32], we found that patients with NHL showed a lower EQ-Index, and a much lower EQ-Index was observed when patients reported having a high financial burden. This supported the findings from a previous study using the SF-12 in an American population, which concluded that financial burden results in a worsened HRQoL for NHL patients who already have poor health [16]. Additionally, we identified that, except for patients with high financial burden, NHL patients who reported suffering low or moderate financial burden were more likely to indicate a higher EQ-Index when using the subjective method than when using the objective method. This finding further suggests the important role that financial burden has in affecting patients’ mental health, and making them feel safe (e.g., strength insurance system) might be more important than directly providing monetary support (e.g., medical voucher). The EQ-Index, as a useful indicator, provides decision makers with a comprehensive perspective to understand the effect of financial burden on physical and mental HRQoL and offers a simple and rapid way to develop a better understanding of how such a relationship may affect NHL patients’ decision-making in cancer-related social welfare systems [33].

In addition to its impact on anxiety and depression, and effects on the overall HRQoL, NHL can reshape patients’ relationships with their families and community. We observed that, compared with the other HRQoL functioning dimensions, financial concerns show more impact on NHL patients’ ability to be involved in social activates. Individuals with NHL often live at risk of psychosocial maladjustment and some other life-long adverse effects or disabilities, and their life is more vulnerable if it is associated with financial hardship, which tends to jeopardize their social relationships [34]. Cancer could incur direct or indirect costs, as well as medical or nonmedical costs, that significantly limit the patients’ ability to participate in social activities [35]. Costa et al. found that family members of patients who suffer financial distress are more likely to spend a longer time working and experience great pressure to control and reduce financial crises, which could limit their time and energy to share emotions with patients or help them engage in community activities [36]. Thus, social support appears to be a key strategy to help patients with NHL or other cancers cope with stress and trauma and communicate with the community to reduce the negative impacts of disease on their HRQoL [37]. NHL patients and their family cannot take on the problem of financial difficulty alone. Policy makers and medical professionals must arm themselves with knowledge about the sources of financial burden of NHL or the other cancers treatment and help to reduce the impact of that burden on patients and their family on the level of both health system and consultation room [38]. In addition, in this study, the effects of long-term financial burden on patients’ social and other functioning were not investigated. For example, the role of resilience, a positive characteristic that supports a patient’s capacity to maintain well-being [36], should be considered in future studies.

We found that after adjusting for the patients’ demographics, SES, and duration of NHL, the differences in HRQoL between different levels of financial burdens were larger than indicated by the results from the unadjusted models. We further divided respondents into two groups based on the commonality of their subtypes (i.e., patients with CLL, FL and DLCBL were defined as having “common NHL”, and the rest was defined as “uncommon NHL”) (Table S5). Multivariable analysis indicated the difference in terms of the impact of financial burden on HRQoL between the “common NHL” and the “uncommon NHL” patient groups was rather small and insignificant (Tables S6 and S7). Yet these findings still shed some light on the financial burden of cancer care needs to be examined in a broader context considering patients’ personal characteristics and their experience with disease [38]. It would be better to take the individual’s heterogeneities into consideration, which may show more implications for explaining the differences in patients’ HRQoL and their ability to cope with NHL. Chen et al. also indicated that it is possible to consider more relative conceptualizations of healthcare cost when evaluating their effect on HRQoL [27]. Furthermore, the effect of duration of NHL and treatment status on patients’ HRQoL were significant and being included in the development of the models. They were identified by previous studies that could affect the relationship between HRQoL and financial burden in some cancer patients [35,39]. However, both of them might not be able to fully reflect the severity of the NHL, and may have reduced the validity of our conclusion, and such clinical information should be directly elicited from the medical records for use in future studies.

Several limitations should be addressed. First, this was a cross-sectional study, and thus, no causal relationship could be determined. Second, the data used in the analyses came from an online survey, which means that patients with very poor health status or patients unable to access the Internet may have been excluded from the survey. Third, recall bias may have affected the self-reported data and reduced the validity of our findings. Fourth, we did not check the patients’ medical records; hence, it is not determined the poor mental HRQoL was caused by either a high financial burden or their own psychological illness. Last, no clinical data about the severity and prognosis of NHL, such as the aggressiveness of underlying hematological malignant disease, were collected through the survey or included in the analyses, which may limit the generalizability of our findings. Such information need to be attended in the future studies.

## 4. Methods

### 4.1. Data Source and Collection

The data used for this analysis come from a nationwide cross-sectional online survey that aimed to examine the impact of healthcare service use, demographic characteristics, financial burden, and social networks on the well-being of patients with lymphomas in China between May and July 2019. The survey was conducted with the assistance of House 086, which is the largest (≥70,000 registered members) patient organization that serves patients with lymphomas in China. In this survey, all the respondents were registered members of House 086. The inclusion criteria were as follows: (1) ≥18 years old, (2) able to read and write Chinese, (3) no cognitive problems, and (4) willing and able to provide informed consent. The research team closely collaborated with House086 to ensure that all the respondents met our criteria. All the respondents were recruited via the House086 internal social media groups that allowed only registered members to participate (WeChat or QQ). Interested members were invited to join in a specific online “survey group.” Subsequently, staff members of the research team and House086 informed the respondents about the study and administered the survey. All the respondents provided informed consent before they participated in the survey. Finally, data from 1549 patients who reported having different types of NHL and provided full information on their financial status were elicited for our analyses. This study was approved by the Institutional Review Board of the Chinese University of Hong Kong (Ref. No.: SBRE-18–268).

### 4.2. Measurement of HRQoL

#### 4.2.1. The 5-Level EQ-5D (EQ-5D-5L)

The EQ-5D-5L Chinese version was adopted in this study to estimate the HRQoL in patients with NHL [40]. The descriptive system of the EQ-5D-5L consists of five dimensions (mobility, self-care, usual activities, pain/discomfort, and anxiety/depression) with five levels (from ”no problem” to ”extreme problem”). All health states (5^5^ = 3125) can be converted into a single health preference index score (EQ-Index). The EQ-Index ranges from 0 (death) to 1 (full health). In this study, the EQ-Index was calculated based on the Chinese population tariff [41].

#### 4.2.2. EORTC QLQ-C30 (QLQ-C30)

The QLQ-C30 is a validated questionnaire comprising 30 items to evaluate quality of life in cancer patients [42]. In this study, the results of three functional subscales (physical, emotional, and social) were elicited to reflect the patients’ HRQoL. The scores of each subscale range from 0 to 100, and a higher score indicates better functioning or HRQoL.

### 4.3. Measurement of Financial Burden

Financial burden was estimated using two methods. Subjective financial burden was evaluated using the item “Financial difficulties” from the QLQ-C30 scale. The question was “*Has your physical condition or medical treatment caused you financial difficulties?*” All the respondents indicated their choice on a four-point Likert scale with the categories of “not at all”, “a little”, “quite a bit”, and “very much”. To estimate the objective financial burden, two steps were followed. In the first step, respondents were asked to make a choice to indicate how much money they have paid for their NHL-related healthcare in the last year from a couple of predefined cost ranges, e.g., 1000~2000, etc. In the second step, respondents were asked to further provide the exact number they have paid for their NHL-related healthcare in the last year, within the cost range they chose in the previous step. Information on four types of healthcare costs was collected: treatment-related cost (diagnosis, treatment, rehabilitation, and medicines); transportation-related cost (transportation, hotel, etc.); caregiver-related cost (caregiver salary, nursing fee, etc.); and miscellaneous cost (nutrition, mobility aids [canes, walkers, etc.]). Patients were asked to repeat the above two steps to estimate all the aforementioned four types of costs, and then the costs of all the types were summed and defined as the overall healthcare cost. The objective financial burden was calculated and reflected by the healthcare cost-to-income ratio (overall healthcare cost/overall family income). Considering that most patients reported no personal income due to NHL, in this study, we used the family income (same procedure, choosing range and then providing exact amount), rather than the patient’s individual income, as the denominator to calculate the objective financial burden. Regarding the levels of objective financial burden, we followed Chen et al.’s method [27], and the cost-to-income ratio was re-categorized into three groups: <40%, 40%~99%, ≥100%, which indicated low, moderate and high objective financial burden. To ensure comparability, we also regrouped the subjective financial burden from four categories into three categories (low [“not at all” and “a little”], moderate [“quite a bit”] and high [“very much”]). Cost data were converted from Chinese Yuan (CNY) to the US Dollar (USD), and the reference exchange rate was 1 USD, equal to 7.017 RMB (December 2019).

### 4.4. Statistical Analysis

Descriptive analysis was used to demonstrate the patients’ demographics, socioeconomic status (SES), health conditions, and HRQoL. The prevalence of financial burden estimated on the basis of subjective and objective methods was also presented. The chi-squared test was used to examine the differences between patients who reported different levels of financial burdens. Kruskal-Wallis one-way analysis of variance was used to compare the HRQoL scores between different levels of financial burden. Ordinal least squares (OLS) and Tobit regression models (a censored regression model to estimate linear relationships between variables when there is either left- or right-censoring in the dependent variable [EQ-Index]) [30] were used to assess the relationship between subjective/objective financial burden and HRQoL (EQ-Index, physical, emotional and social functioning) adjusted by respondents’ demographics (sex, age), SES (educational level, family registry), treatment regimens (chemotherapy and radiation therapy), treatment status, and duration of NHL. R (R Foundation, Austria) was used to conduct all the data analysis. Bonferroni correction was employed in our analysis, and the *p*-value was set at 0.05/35 = 0.001.

## 5. Conclusions

The results of the current study demonstrated that nearly 60% of patients with NHL suffer a moderate-to-high cancer-related financial burden in China. Increased financial burden resulted in poorer HRQoL and particularly jeopardized the patients’ social functioning. Controlling the financial burden for patients with NHL is a complicated process that requires integrated efforts from all stakeholders, including patients, healthcare professionals, and policy makers. In China, currently, few policies and insurance plans exist to provide full protection to patients with rare diseases, such as some subtypes of NHL. Medical professionals should carefully consider selecting highly cost-effective treatments for patients and engaging them in decision-making to ensure that they understand the potential financial consequences of the options.

## Figures and Tables

**Table 1:** Degree to financial burden by patients’ characteristics (*n* = 1549).

Patients’ Characteristics	n (%)								
	Overall	Subjective Financial Burden				Objective Financial Burden			
		Low	Moderate	High	*p*-Value	Low	Moderate	High	*p*-Value
Sex									
Male	808 (52.2)	297 (36.8)	238 (29.5)	273 (33.8)	0.078	353 (43.7)	129 (16.0)	326 (40.3)	0.254
Female	741 (47.8)	314 (42.4)	199 (26.9)	228 (30.8)		304 (41.0)	141 (19.0)	296 (39.9)	
Age									
≤30	207 (13.4)	71 (34.3)	53 (25.6)	83 (40.1)	0.267	86 (41.5)	27 (13.0)	94 (45.4)	0.218
31–40	503 (32.5)	201 (40.0)	138 (27.4)	164 (32.6)		217 (43.1)	87 (17.3)	199 (39.6)	
41–50	430 (27.8)	171 (39.8)	121 (28.1)	138 (32.1)		184 (42.8)	79 (18.4)	167 (38.8)	
51–60	265 (17.1)	110 (41.5)	80 (30.2)	75 (28.3)		102 (38.5)	57 (21.5)	106 (40.0)	
≥61	141 (9.1)	58 (41.1)	45 (31.9)	38 (27.0)		68 (48.2)	18 (12.8)	55 (39.0)	
Educational attainment									
No/Primary	207 (13.4)	49 (23.7)	48 (23.2)	110 (53.1)	<0.001	59 (28.5)	29 (14.0)	119 (57.5)	<0.001
Secondary/Post-secondary	699 (45.1)	223 (31.9)	212 (30.3)	264 (37.8)		272 (38.9)	125 (17.9)	302 (43.2)	
Tertiary or above	643 (41.5)	339 (52.7)	177 (27.5)	127 (19.8)		326 (50.7)	116 (18.0)	201 (31.3)	
Health Insurance ^									
UEBMI/URBMI	1165 (75.2)	128 (33.3)	94 (24.5)	162 (42.2)	<0.001	511 (43.9)	211 (18.1)	443 (38.0)	<0.001
NCMS	203 (13.1)	36 (17.7)	52 (25.6)	115 (56.7)		64 (31.5)	28 (13.8)	111 (54.7)	
CIS	94 (6.1)	64 (68.1)	21 (22.3)	9 (9.6)		54 (57.4)	16 (17.0)	24 (25.5)	
No insurance	12 (0.8)	3 (25.0)	3 (25.0)	6 (50.0)		2 (16.7)	0	10 (83.3)	
Family register									
Urban residents	1170 (75.5)	513 (43.8)	332 (28.4)	325 (27.8)	<0.001	529 (45.2)	209 (17.9)	432 (36.9)	<0.001
Rural residents	372 (24.5)	92 (24.7)	105 (28.2)	175 (47.0)		124 (33.3)	60 (16.1)	188 (50.5)	
Duration of disease (years)									
1–3	785 (58.5)	310 (39.5)	223 (28.4)	252 (32.1)	0.552	203 (25.9)	145 (18.5)	437 (55.7)	<0.001
4–6	395 (29.5)	174 (44.1)	93 (23.5)	128 (32.4)		215 (54.4)	76 (19.2)	104 (26.3)	
7–10	120 (9.0)	47 (39.2)	34 (28.3)	39 (32.5)		70 (58.3)	19 (15.8)	31 (25.8)	
≥10	40 (3.0)	17 (42.5)	13 (32.5)	10 (25.0)		24 (60.0)	5 (12.5)	11 (27.5)	
Family income (USD/year)									
≤20000	496 (51.3)	199 (40.1)	151 (30.4)	146 (29.4)	<0.001	198 (39.9)	87 (17.5)	211 (42.5)	<0.001
20001–30000	268 (27.7)	152 (56.7)	77 (28.7)	39 (14.6)		146 (54.5)	54 (20.1)	68 (25.4)	
30001–50000	115 (11.9)	77 (67.0)	23 (20.0)	15 (13.0)		72 (62.6)	28 (24.3)	15 (13.0)	
≥50001	88 (9.1)	72 (81.8)	11 (12.5)	5 (5.7)		75 (85.2)	12 (13.6)	1 (1.1)	
Chemotherapy									
Yes	1292 (90.2)	487 (37.7)	356 (27.6)	449 (34.8)	0.009	489 (37.8)	233 (18.0)	570 (44.1)	<0.001
No	141 (9.8)	65 (46.1)	45 (31.9)	31 (22.0)		78 (55.3)	22 (15.6)	41 (29.1)	
Immunotherapy									
Yes	609 (42.5)	241 (39.6)	176 (28.9)	192 (31.5)	0.396	244 (40.1)	105 (17.2)	260 (42.7)	0.882
No	824 (57.5)	311 (37.7)	225 (27.3)	288 (35.0)		323 (39.2)	150 (18.2)	351 (42.6)	
Radiation therapy									
Yes	308 (21.5)	140 (45.5)	79 (25.6)	89 (28.9)	0.017	109 (35.4)	61 (19.8)	138 (44.8)	0.217
No	1125 (78.5)	412 (36.6)	322 (28.6)	391 (34.8)		458 (40.7)	194 (17.2)	473 (42.0)	
Surgery									
Yes	221 (15.4)	80 (36.2)	59 (26.7)	82 (37.1)	0.46	94 (42.5)	32 (14.5)	95 (43.0)	0.332
No	1212 (84.6)	472 (38.9)	342 (28.2)	398 (32.8)		473 (39.0)	223 (18.4)	516 (42.6)	
Treatment status									
Never be treated (no need for treatment)	116 (7.5)	59 (50.9)	36 (31.0)	21 (18.1)	<0.001	90 (77.6)	15 (12.9)	11 (9.5)	<0.001
Currently not undertreatment (improved due to last treatment)	122 (7.9)	59 (48.4)	26 (21.3)	37 (30.3)		70 (57.4)	20 (16.4)	32 (26.2)	
Under treatment	612 (39.5)	180 (29.4)	197 (32.2)	235 (38.4)		240 (39.2)	117 (19.1)	255 (41.7)	
Treatment just completed	699 (45.1)	313 (44.8)	178 (25.5)	208 (29.8)		257 (36.8)	118 (16.9)	324 (46.4)	
Sub-types									
CLL	158 (10.2)	66 (41.8)	50 (31.6)	42 (26.6)	<0.001	98 (62.0)	27 (17.7)	33 (20.9)	<0.001
FL	359 (23.2)	149 (41.5)	106 (29.5)	104 (29.0)		159 (44.3)	76 (21.2)	124 (34.5)	
LPL	31 (2.0)	12 (38.7)	9 (29.0)	10 (32.3)		17 (54.8)	3 (9.7)	11 (35.5)	
MALT	86 (5.6)	42 (48.8)	22 (25.6)	22 (25.6)		41 (47.7)	16 (18.6)	29 (33.7)	
MCL	76 (4.9)	10 (13.2)	22 (28.9)	44 (57.9)		23 (30.3)	17 (22.4)	36 (47.4)	
DLBCL	583 (37.6)	249 (42.7)	159 (27.3)	175 (30.0)		238 (40.8)	91 (15.6)	254 (43.6)	
BL	27 (1.7)	6 (22.2)	8 (29.6)	13 (48.1)		11 (40.7)	2 (7.4)	14 (51.9)	
T-LBL	35 (2.3)	7 (20.0)	6 (17.1)	22 (62.9)		9 (25.7)	4 (11.4)	22 (62.9)	
ENKL	114 (7.4)	43 (37.7)	32 (28.1)	39 (34.2)		38 (33.3)	23 (20.2)	53 (46.5)	
PTCL	34 (2.2)	11 (32.4)	10 (29.4)	13 (38.2)		3 (8.8)	6 (17.6)	25 (73.5)	
PC-ALCL	46 (3.0)	16 (34.8)	13 (28.3)	17 (37.0)		20 (43.5)	5 (17.4)	21 (40.2)	

UEBMI, urban employee basic medical insurance; URBMI, urban resident basic medical insurance; NCMS, New rural cooperative medical scheme; CIS, commercial insurance scheme. ^ 75 respondents did not report. CLL, chronic lymphocytic leukemia; FL, Follicular lymphoma; LPL, Lymphoplasmacytic lymphoma; MALT, Mucosa associated lymphoid tissue type; MCL, Mantle cell lymphoma; DLBCL, Diffuse large B cell lymphoma; BL, Burkitt lymphoma; T-LBL, T-cell lymphoblastic *lymphoma*; ENKL, Extranodal natural killer (*NK*)/*T*-*cell lymphoma; PTCL,* peripheral T cell lymphoma; PC-ALCL, primary cutaneous anaplastic large cell lymphoma.

**Table 2:** Comparison between patients reported subjective and objective financial burden.

Objective Financial Burden (Cost-to-Income Ratio)	Subjective Financial Burden				
	Low	Moderate	High	Overall	*p*-Value
Overall	611 (39.4)	437 (28.2)	501 (32.3)	1549 (100)	
Low (<40%)	342	178	137	657 (42.4)	<0.001
Moderate (40%~100%)	104	77	89	270 (17.4)	
High (>100%)	165	182	275	622 (40.2)	
Male	297 (36.8)	238 (29.5)	273 (33.8)	808 (100)	
Low (<40%)	167	103	83	353 (43.7)	<0.001
Moderate (40%~100%)	49	42	38	129 (16.0)	
High (>100%)	81	93	152	326 (40.3)	
Female	314 (42.4)	199 (26.9)	228 (30.8)	741 (100)	
Low (<40%)	175	75	54	304 (41.0)	<0.001
Moderate (40%~100%)	55	35	51	141 (19.0)	
High (>100%)	84	89	123	296 (40.0)	

**Table 3:** Patients’ HRQoL stratified by financial burden and treatment status.

	Mean (Standard Deviation)								
Treatment Status	Objective Financial Burden				Subjective Financial Burden				Overall
	Low	Moderate	High	*p*-Value	Low	Moderate	High	*p*-Value	Mean (sd)
Overall									
EQ Index	0.89 (0.12)	0.85 (0.16)	0.84 (0.19)	<0.001	0.92 (0.09)	0.87 (0.13)	0.79 (0.21)	<0.001	0.86 (0.16)
Physical functioning	83.8 (15.4)	78.2 (17.7)	77.1 (18.4)	<0.001	86.9 (13.4)	79.9 (14.8)	71.9 (19.9)	<0.001	80.1 (17.3)
Emotional functioning	68.7 (22.2)	63.9 (24.9)	64.3 (24.2)	<0.001	77 (17.6)	65.9 (19.5)	53 (26.4)	<0.001	66.1 (23.6)
Social functioning	56.2 (30)	47.7 (29.7)	43.3 (28.8)	<0.001	70.7 (22)	47.9 (22.2)	25.1 (25.2)	<0.001	49.5 (30.1)
Minor									
EQ Index	0.91 (0.1)	0.87 (0.1)	0.87 (0.2)	0.27	0.93 (0.08)	0.9 (0.08)	0.8 (0.13)	<0.001	0.9 (0.11)
Physical functioning	88.5 (11.8)	82.2 (11.82)	80.6 (13.8)	0.01	90.6 (10.4)	85.4 (11.8)	79.4 (13.8)	<0.001	87 (12.2)
Emotional functioning	70.2 (20.9)	65 (23)	59.8 (34.3)	0.59	77.8 (15.5)	65.3 (19.8)	48 (29.3)	<0.001	68.5 (22.7)
Social functioning	68.7 (27.2)	62.2 (27.8)	36.4 (35.6)	0.01	81.6 (19.3)	58.3 (18.9)	28.6 (31.7)	<0.001	64.8 (29.5)
Moderate									
EQ Index	0.89 (0.1)	0.84 (0.1)	0.87 (0.1)	0.11	0.91 (0.08)	0.84 (0.13)	0.84 (0.11)	<0.001	0.88 (0.11)
Physical functioning	85.8 (14.9)	73 (15.7)	79.4 (18.8)	0.002	86.2 (15.6)	81 (16.2)	76 (17.3)	0.002	82 (16.7)
Emotional functioning	69.1 (23.7)	55 (26.8)	72.9 (24.6)	0.02	76.1 (18.6)	69.9 (22.5)	52.9 (29)	<0.001	67.8 (25)
Social functioning	58.8 (30.3)	45 (30.2)	53.6 (30.7)	0.15	73.5 (21)	52.6 (26.5)	27.9 (24.9)	<0.001	55.2 (30.5)
Severe									
EQ Index	0.86 (0.15)	0.81 (0.19)	0.78 (0.22)	<0.001	0.89 (0.12)	0.85 (0.13)	0.73 (0.25)	<0.001	0.82 (0.19)
Physical functioning	78 (17.4)	73.2 (19.3)	71.7 (20.3)	<0.001	82.4 (16)	76.4 (15.5)	66.8 (21.3)	<0.001	74.5 (19.2)
Emotional functioning	65.2 (21.6)	60.3 (25.9)	58 (24)	0.001	73.9 (16.3)	64.3 (19.4)	49 (25.4)	<0.001	61.3 (23.5)
Social functioning	45.1 (27.3)	41.9 (30.2)	36.7 (26.8)	0.004	63.4 (20.2)	45.2 (20.9)	20.4 (22.9)	<0.001	41 (27.9)
Treatment completed									
EQ Index	0.92 (0.1)	0.9 (0.12)	0.89 (0.14)	0.006	0.94 (0.07)	0.89 (0.14)	0.85 (0.16)	<0.001	0.9 (0.13)
Physical functioning	87 (13.1)	83.5 (15.3)	80.9 (15.7)	<0.001	89 (11.1)	82.6 (13.6)	76.3 (17.7)	<0.001	83.6 (15)
Emotional functioning	71.5 (22.5)	68.7 (23)	68.6 (23)	0.2	78.7 (18.3)	67.3 (19.1)	58.1 (26)	<0.001	69.7 (22.8)
Social functioning	61.4 (30.3)	52.1 (28.4)	47.7 (29)	<0.001	72.3 (22.4)	48.1 (23)	29.7 (26.4)	<0.001	53.5 (30)

Note: Minor: Never be treated (no need for treatment); Moderate: Currently not under treatment (improved due to last treatment); Severe: Under treatment; Treatment completed: Treatment just completed; sd, Standard deviation.

**Table 4:** Multivariable adjusted linear regression models of the relationship between HRQoL and financial burdens.

	b (95% C.I.)			
Financial Burdens	EQ Index	Physical Functioning	Emotional Functioning	Social Functioning
Objective burden				
< 40%	Ref	Ref	Ref	Ref
40%~100%	−0.03 (−0.06~0.002)	−4.0 (−6.5~ −1.6) **	−4.4 (−7.7~ −1.1) *	−6.2 (−10.4~ −1.9) **
> 100%	−0.04 (−0.07~ −0.02) ***	−5.3 (−7.3~ −3.4) ***	−4.1 (−7~ −1.6) **	−8.8 (−12.1~ −5.4) ***
Subjective burden				
Low	Ref	Ref	Ref	Ref
Moderate	−0.07 (−0.1~ −0.05) ***	−6.1 (−8.1~ −4.1) ***	−10.8 (−13.6~ −8.0) ***	−21.4 (−24.4~ −18.5) ***
High	−0.15 (−0.17~ −0.13) ***	−14.4 (−16.4~ −12.4) ***	−24.4 (−27.1~ −21.6) ***	−43.6 (−46.6~ −40.7) ***

Note: models were adjusted by sex, age, educational level, family registration, treatment status, chemotherapy, radiation therapy, and duration; * *p* < 0.05; ** *p* < 0.01; *** *p* < 0.001. b, coefficient; 95% C.I., 95% confidence interval; For EQ-Index, Tobit regression model was used; for PF, EF and SF, the OLS model was used.

## Data Availability

The data that support the findings of this study are available from the corresponding author upon reasonable request.

## Supplementary Materials

The following are available online at https://www.mdpi.com/2072-6694/12/11/3325/s1, Table S1: Multivariable adjusted linear regression models of the relationship between EQ-Index and financial burdens, Table S2: Multivariable adjusted linear regression models of the relationship between physical functioning and financial burdens, Table S3: Multivariable adjusted linear regression models of the relationship between emotional functioning and financial burdens, Table S4: Multivariable adjusted linear regression models of the relationship between social functioning and financial burdens, Table S5: Comparisons between common and uncommon NHL, Table S6: Multivariable adjusted linear regression models of the relationship between HRQoL and financial burdens for patients with common NHL, Table S7: Multivariable adjusted linear regression models of the relationship between HRQoL and financial burdens for patients with uncommon NHL.
