# Supplementary material for: Quantifying the Effect of Financial Burden on Health-Related Quality of Life among Patients with Non-Hodgkin’s Lymphomas
Source: Cancers (Basel). 2020 Nov 11;12(11):3325. doi: 10.3390/cancers12113325 (PMC7698092; doi:10.3390/cancers12113325)
Supplement: Supplementary file 1 [file cancers-12-03325-s001.pdf]

cancers

Article

Quantifying the Effect of Financial Burden on Health-Related Quality of Life among Patients with Non-Hodgkin's Lymphomas

Richard Huan Xu [1,2], Eliza Lai-yi Wong [1,2], Yi Su [3], Hongyu Zhang [4], Wei Zhang [5] and Dong Dong [1,2,6,*]

1 Jockey Club School of Public Health and Primary Care, Faculty of Medicine, The Chinese University of Hong Kong, Hong Kong SAR, China; richardhxu@cuhk.edu.hk (R.H.X.); lywong@cuhk.edu.hk (E.L.W.)
2 Centre for Health Systems and Policy Research, The Chinese University of Hong Kong, Hong Kong SAR, China
3 Department of Health Affairs, Jinling Hospital, School of Medicine, Nanjing University, Nanjing 210000, China; sy_njzy@126.com
4 Department of Hematology, Peking University Shenzhen Hospital, Shenzhen, Guangdong, 518000 China; hongyuzhang@pkusz.com
5 Department of Hematology, Peking Union Medical College Hospital, Beijing, 100730 China; vv1223@vip.sina.com
6 Shenzhen Research Institute, The Chinese University of Hong Kong, Shenzhen, 518000 Guangdong, China
* Correspondence: dongdong@cuhk.edu.hk; Tel.: +852-2252-8461; Fax: +852-2606-3500

Received: date; Accepted: date; Published: date

Supplementary

Table S1. Multivariable adjusted linear regression models of the relationship between EQ-Index and financial burdens.

**Coefficients:**	**Objective**					**Subjective**				
	Estimate	Std. Error	z value	Pr(>|z|)		Estimate	Std. Error	z value	Pr(>|z|)	
(Intercept)	1.047918	0.065629	15.967	< 2e-16	***	1.132332	0.062745	18.046	< 2e-16	***
sex	-0.02989	0.010321	-2.896	0.00378	**	-0.03483	0.009781	-3.561	0.000369	***
Age	-0.00185	0.000515	-3.584	0.000338	***	-0.00247	0.000491	-5.031	4.87E-07	***
hukou	0.005514	0.012783	0.431	0.666229		0.008737	0.01212	0.721	0.471012	
edusecondary	0.017024	0.016173	1.053	0.292511		0.006204	0.015337	0.405	0.685825	
edutertiary	0.040839	0.017459	2.339	0.019324	*	0.001857	0.016802	0.111	0.912009	
duration	0.001604	0.002083	0.77	0.44138		0.002926	0.001958	1.494	0.135183	
as.factor(treat)3	-0.07124	0.02245	-3.173	0.001507	**	-0.05863	0.021325	-2.749	0.005975	**
as.factor(treat)4	0.033394	0.022158	1.507	0.13178		0.026208	0.020967	1.25	0.211319	
chemo	-0.0387	0.040286	-0.961	0.336726		-0.02649	0.038145	-0.694	0.487407	
radio	-0.00797	0.012878	-0.619	0.536058		-0.02166	0.012228	-1.771	0.07649	.
Moderate burden	-0.02672	0.014685	-1.819	0.068851	.	-0.07373	0.012408	-5.942	2.81E-09	***
Severe burden	-0.04412	0.011714	-3.766	0.000166	***	-0.15355	0.012143	-12.645	< 2e-16	***
Log(scale)	-1.71805	0.02283	-75.254	< 2e-16	***	-1.77376	0.022747	-77.977	< 2e-16	***

Table S2. Multivariable adjusted linear regression models of the relationship between physical functioning and financial burdens.

**Coefficients:**	**Objective**					**Subjective**				
	Estimate	Std. Error	t value	Pr(>|t|)		Estimate	Std. Error	t value	Pr(>|t|)	
(Intercept)	103.4344	4.35467	23.753	< 2e-16	***	111.3542	4.1712	26.696	< 2e-16	***
sex	-4.80128	0.87457	-5.49	4.76E-08	***	-5.45821	0.82827	-6.59	6.19E-11	***
Age	-0.23458	0.04349	-5.394	8.06E-08	***	-0.29558	0.04133	-7.151	1.38E-12	***
hukou	0.55945	1.09854	0.509	0.610648		0.88866	1.04043	0.854	0.39318	
edusecondary	0.30931	1.39261	0.222	0.824263		-0.78682	1.32059	-0.596	0.5514	
edutertiary	2.41447	1.50101	1.609	0.107934		-1.0484	1.4425	-0.727	0.46747	
duration	0.25644	0.17323	1.48	0.139008		0.42586	0.16258	2.619	0.0089	**
as.factor(treat)3	-5.9517	1.69299	-3.515	0.000453	***	-5.14017	1.60766	-3.197	0.00142	**
as.factor(treat)4	2.87658	1.69746	1.695	0.090363	.	1.85079	1.60322	1.154	0.24852	
chemo	-6.36473	1.517	-4.196	2.89E-05	***	-5.73129	1.4353	-3.993	6.86E-05	***
radio	1.77794	1.08733	1.635	0.102241		0.40853	1.03156	0.396	0.69214	
Moderate burden	-4.01635	1.2411	-3.236	0.00124	**	-6.10335	1.03402	-5.903	4.47E-09	***
Severe burden	-5.32395	0.98837	-5.387	8.39E-08	***	-14.3978	1.02689	-14.021	< 2e-16	***

Table S3. Multivariable adjusted linear regression models of the relationship between emotional functioning and financial burdens.

**Coefficients:**	**objective**					**Subjective**				
	Estimate	Std. Error	t value	Pr(>|t|)		Estimate	Std. Error	t value	Pr(>|t|)	
(Intercept)	64.40455	6.16987	10.439	< 2e-16	***	79.72836	5.68555	14.023	< 2e-16	***
sex	-3.66056	1.23912	-2.954	0.00319	**	-4.67769	1.12898	-4.143	3.63E-05	***
Age	0.12849	0.06162	2.085	0.03722	*	0.03158	0.05634	0.561	0.57519	
hukou	3.2229	1.55646	2.071	0.03857	*	3.97148	1.41816	2.8	0.00517	**
edusecondary	0.87401	1.97311	0.443	0.65786		-1.47167	1.80003	-0.818	0.41373	
edutertiary	3.46112	2.12669	1.627	0.10386		-3.42777	1.9662	-1.743	0.08149	.
duration	-0.12477	0.24544	-0.508	0.6113		0.04542	0.2216	0.205	0.83764	
as.factor(treat)3	-5.50182	2.3987	-2.294	0.02196	*	-3.57939	2.19132	-1.633	0.1026	
as.factor(treat)4	3.12084	2.40503	1.298	0.19462		2.11052	2.18526	0.966	0.33431	
chemo	-1.35092	2.14935	-0.629	0.52976		0.2779	1.95639	0.142	0.88706	
radio	2.57029	1.54057	1.668	0.09546	.	0.41875	1.40607	0.298	0.76589	
Moderate burden	-4.45436	1.75843	-2.533	0.01141	*	-10.8021	1.40942	-7.664	3.31E-14	***
Severe burden	-4.12957	1.40036	-2.949	0.00324	**	-24.3851	1.3997	-17.422	< 2e-16	***

Table S4. Multivariable adjusted linear regression models of the relationship between social functioning and financial burdens.

**Coefficients:**	**Objective**					**Subjective**				
	Estimate	Std. Error	t value	Pr(>|t|)		Estimate	Std. Error	t value	Pr(>|t|)	
(Intercept)	54.79279	7.57315	7.235	7.58E-13	***	81.87328	6.13428	13.347	< 2e-16	***
sex	0.85744	1.52095	0.564	0.573011		-0.95241	1.21808	-0.782	0.43441	
Age	0.10053	0.07563	1.329	0.184016		-0.06679	0.06079	-1.099	0.27208	
hukou	-1.48353	1.91046	-0.777	0.437565		-0.15029	1.53008	-0.098	0.92177	
edusecondary	1.27611	2.42187	0.527	0.598339		-2.48352	1.9421	-1.279	0.20118	
edutertiary	8.26745	2.61039	3.167	0.001572	**	-3.46491	2.12138	-1.633	0.10262	
duration	0.2569	0.30127	0.853	0.393947		0.57716	0.23909	2.414	0.0159	*
as.factor(treat)3	-9.71977	2.94427	-3.301	0.000986	***	-6.10581	2.36427	-2.583	0.00991	**
as.factor(treat)4	2.96923	2.95203	1.006	0.31467		1.08865	2.35773	0.462	0.64434	
chemo	-9.34978	2.6382	-3.544	0.000407	***	-6.60694	2.11079	-3.13	0.00178	**
radio	3.99461	1.89097	2.112	0.034821	*	0.16511	1.51704	0.109	0.91335	
Moderate burden	-6.19134	2.15838	-2.869	0.004185	**	-21.4389	1.52066	-14.098	< 2e-16	***
Severe burden	-8.78699	1.71886	-5.112	3.62E-07	***	-43.6504	1.51017	-28.904	< 2e-16	***

Table S5. Comparisons between common and Uncommon NHL.

	**Common NHL n=1100**	**Uncommon NHL n=449**	**p-value**
EQ-Index	0.87(0.15)	0.85(0.19)	<0.001
Physical function	78.25(18.59)	66.08(23.95)	<0.001
Emotional function	66.08(23.95)	66.22(22.65)	0.23
Social function	51.29(30.05)	45.25(29.75)	<0.001
Subjective burden			
Low	464(42.2)	147(32.7)	<0.001
Moderate	315(28.6)	122(27.2)	
High	321(29.2)	180(40.1)	
Objective burden			
Low	495(45.0)	162(36.1)	0.001
Moderate	194(17.6)	76(16.9)	
High	411(37.4)	211(47.0)	

Common NHL: CLL, FL and DLBCL.Uncommon NHL: LPL, MALT, MCL, BL, T-LBL, ENKL, PTCL, and PC-ALCL

Table S6. Multivariable adjusted linear regression models of the relationship between HRQoL and financial burdens for patients with common NHL.

	**b (95% C.I.)**			
	**EQ Index**	**Physical Functioning**	**Emotional Functioning**	**Social Functioning**
Objective method				
<40%	Ref	Ref	Ref	Ref
40%~100%	-0.04(-0.07~-0.01)***	-4.75(-7.5~-1.97)***	-5.39(-9.54~-1.22)*	-5.37(-10.4~-0.34)*
>100%	-0.05(-0.07~-0.02)***	-3.94(-6.19~-1.68)***	-5.07(-8.44~-1.71)**	-8.27(-12.34~-4.19)***
Subjective method				
Low	Ref	Ref	Ref	Ref
Moderate	-0.07(-0.1~-0.05)***	-6.43(-8.73~-4.13)***	-11.26(-14.51~-8.01)***	-20.99(-24.54~-17.45)***
High	-0.15(-0.18~-0.13)***	-14.22(-16.55~-11.87)***	-27.35(-30.66~-24.04)***	-43.25(-46.86~-39.64)***

cancers

MDPI

Table S7. Multivariable adjusted linear regression models of the relationship between HRQoL and financial burdens for patients with uncommon NHL.

	b (95% C.I.)			
	**EQ Index**	**Physical Functioning**	**Emotional Functioning**	**Social Functioning**
Objective method				
<40%	Ref	Ref	Ref	Ref
40%~100%	0.02(-0.04~0.09)	-1.55(-6.47~3.36)	-1.68(-7.97~4.6)	-7.53(-15.48~0.43)
>100%	-0.03(-0.08~0.02)	-7.28(-11.06~-3.49)***	-1.92(-6.76~2.9)	-8.21(-14.33~-2.09)**
Subjective method				
Low	Ref	Ref	Ref	Ref
Moderate	-0.07(-0.12~-0.1)***	-4.97(-9.2~-0.75)*	-9.88(-15.14~-4.61)***	-22.48(-28.08~-16.88)***
High	-0.15(-0.2~-0.1)***	-13.91(-17.97~-9.84)***	-19.24(-24.3~-14.18)***	-44.54(-49.93~-39.15)***
